# 2-(Benzo[*d*]thia­zol-2-ylsulfan­yl)-*N*-(6-methyl-2-pyrid­yl)acetamide

**DOI:** 10.1107/S1600536809011519

**Published:** 2009-04-02

**Authors:** Bing Zhao, Hui Wang, Qiang Li, Yan Gao, Dong Liang

**Affiliations:** a Chemistry and Chemical Engineering Institute, Qiqihar University, Heilongjiang, Qiqihar 161006, People’s Republic of China; b Hermann Gmeiner Vocational Technical College, Qiqihar University, Heilongjiang, Qiqihar 161006, People’s Republic of China; c School of Chemical Engineering, University of Science and Technology, Liaoning Anshan, 114051, People’s Republic of China

## Abstract

In the title compound, C_15_H_13_N_3_OS_2_, the pyridine ring and the benzo[*d*]thia­zole unit subtend a dihedral angle of 57.7 (2)°. The length of the C_*sp*_^2^—S bond [1.7462 (17) Å] is significantly shorter than that of the C_*sp*_^3^—S bond [1.8133 (18) Å]. The crystal structure is stabilized by intra­molecular N—H⋯N and inter­molecular C—H⋯O and C—H⋯N hydrogen-bond inter­actions. Furthermore, C—H⋯π inter­actions stabilize the crystal packing.

## Related literature

For biologically active compounds containing the acylamide system, see: Bennasar *et al.* (2006 ▶); Ladziata *et al.* (2006 ▶). For bond-length data, see: Gao *et al.* (2007 ▶).
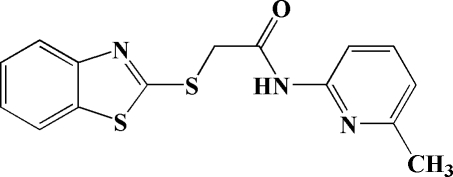


## Experimental

### 

#### Crystal data

C_15_H_13_N_3_OS_2_
*M* *_r_* = 315.40
Triclinic, 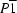

*a* = 8.1919 (16) Å
*b* = 9.0818 (18) Å
*c* = 11.107 (2) Å
α = 74.78 (3)°
β = 89.55 (3)°
γ = 69.34 (3)°
*V* = 742.8 (3) Å^3^
*Z* = 2
Mo *K*α radiation
μ = 0.36 mm^−1^
*T* = 113 K
0.16 × 0.14 × 0.10 mm

#### Data collection

Rigaku Saturn diffractometer
Absorption correction: multi-scan (*SADABS*; Sheldrick, 1996 ▶) *T*_min_ = 0.945, *T*_max_ = 0.965
9391 measured reflections
3526 independent reflections
2615 reflections with *I* > 2σ(*I*)
*R*_int_ = 0.048

#### Refinement

*R*[*F* ^2^ > 2σ(*F* ^2^)] = 0.037
*wR*(*F* ^2^) = 0.092
*S* = 0.99
3526 reflections
195 parameters
H atoms treated by a mixture of independent and constrained refinement
Δρ_max_ = 0.24 e Å^−3^
Δρ_min_ = −0.38 e Å^−3^

### 

Data collection: *CrystalClear* (Molecular Structure Corporation & Rigaku, 1999 ▶); cell refinement: *CrystalClear*; data reduction: *CrystalClear*; program(s) used to solve structure: *SHELXS97* (Sheldrick, 2008 ▶); program(s) used to refine structure: *SHELXL97* (Sheldrick, 2008 ▶); molecular graphics: *SHELXTL* (Sheldrick, 2008 ▶); software used to prepare material for publication: *SHELXTL*.

## Supplementary Material

Crystal structure: contains datablocks global, I. DOI: 10.1107/S1600536809011519/at2753sup1.cif

Structure factors: contains datablocks I. DOI: 10.1107/S1600536809011519/at2753Isup2.hkl

Additional supplementary materials:  crystallographic information; 3D view; checkCIF report

## Figures and Tables

**Table 1:** Hydrogen-bond geometry (Å, °)

*D*—H⋯*A*	*D*—H	H⋯*A*	*D*⋯*A*	*D*—H⋯*A*
N2—H2*A*⋯N3	0.867 (18)	2.142 (18)	2.949 (2)	154.6 (16)
C2—H2⋯O1	0.95	2.30	2.890 (2)	119
C8—H8*A*⋯O1^i^	0.99	2.31	3.239 (2)	156
C8—H8*B*⋯N3	0.99	2.47	2.905 (2)	106
C12—H12⋯N1^ii^	0.95	2.57	3.498 (2)	166
C8—H8*B*⋯*Cg*2^iii^	0.99	2.68	3.494 (2)	140

Symmetry codes: (i) 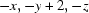; (ii) 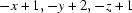; (iii) 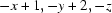. *Cg*2 is the centroid of the N1/C1–C5 ring.

**Fig. 1.**
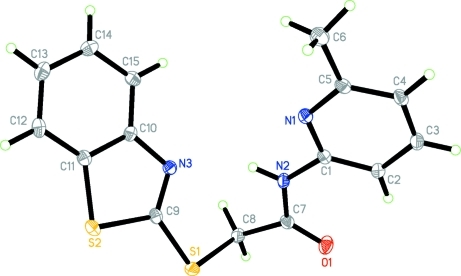
View of the molecule of (I) showing the atom-labeling scheme. Displacement ellipsoids are drawn at the 35% probability level.

## supplementary crystallographic information

### Comment

The acylamide compound is an important class of medical intermediate. Recently, many biological compounds containing acylamide have been reported (Ladziata *et al.*, 2006; Bennasar *et al.*, 2006). Now, we have synthesized the title compound, (I), from the benzo[*d*]thiazole-2-thiol with 6-methylpyridine carbamic chloride. Here, we report its crystal structure.

The molecular structure of (I) and the atom-numbering scheme are shown in Fig. 1. The molecule contains a pyridine ring and a benzo[*d*]thiazole ring. The dihedral angle between the benzene ring and benzo[*d*]thiazole ring is 57.7 (2)°. The methyl carbon attached to the pyridine ring is coplanar to the pyridine ring with an r.m.s deviation of 0.0064 (3) Å. The C1—N1—C7—C8 torsion angle of 178.66 (15)° indicates that the acylamide group are nearly coplanar with the pyridine ring plane. As a result of π-π conjugation, the C*_sp_*^2^—S bond [S1—C9 = 1.7462 (17) Å] is significantly shorter than the C*_sp_*^3^—S bond [S1—C8 = 1.8133 (18) Å]. These values compare with the values of 1.772 (3) and 1.801 (2) Å reported in the literature (Gao *et al.*, 2007). The crystal structure is stabilized by the intramolecular N—H···N and intermolecular C—H···O and C—H···N hydrogen bond interactions. Furthermore, C—H···π interactions stabilize the crystal packing (Table 1).

### Experimental

The title compound was synthesized by the reaction of from the benzo[*d*]thiazole-2-thiol with 6-methylpyridine carbamic chloride in the refluxing ethanol. Crystals of (I) suitable for single-crystal X-ray analysis were grown by slow evaporation of a solution in chloroform/acetone.

### Refinement

The H atom attached to N atom was located in a different density map and the atomic coordinates allowed to refine freely. Other H atoms were positioned geometrically and refined as riding (C—H = 0.95–0.99 Å) and allowed to ride on their parent atoms, with *U*_iso_(H) =1.2*U*_eq_(parent) or 1.5*U*_eq_(parent).

### Crystal data

**Table:**

C_15_H_13_N_3_OS_2_	*Z* = 2
*M**_r_* = 315.40	*F*(000) = 328
Triclinic, *P*1	*D*_x_ = 1.410 Mg m^−^^3^
Hall symbol: -P 1	Mo *K*α radiation, λ = 0.71073 Å
*a* = 8.1919 (16) Å	Cell parameters from 2582 reflections
*b* = 9.0818 (18) Å	θ = 1.9–27.9°
*c* = 11.107 (2) Å	µ = 0.36 mm^−^^1^
α = 74.78 (3)°	*T* = 113 K
β = 89.55 (3)°	Prism, colourless
γ = 69.34 (3)°	0.16 × 0.14 × 0.10 mm
*V* = 742.8 (3) Å^3^	

### Data collection

**Table:**

Rigaku Saturn diffractometer	3526 independent reflections
Radiation source: rotating anode	2615 reflections with *I* > 2σ(*I*)
confocal	*R*_int_ = 0.048
ω scans	θ_max_ = 27.9°, θ_min_ = 1.9°
Absorption correction: multi-scan (*SADABS*; Sheldrick, 1996)	*h* = −10→10
*T*_min_ = 0.945, *T*_max_ = 0.965	*k* = −11→11
9391 measured reflections	*l* = −14→14

### Refinement

**Table:**

Refinement on *F*^2^	Primary atom site location: structure-invariant direct methods
Least-squares matrix: full	Secondary atom site location: difference Fourier map
*R*[*F*^2^ > 2σ(*F*^2^)] = 0.037	Hydrogen site location: inferred from neighbouring sites
*wR*(*F*^2^) = 0.092	H atoms treated by a mixture of independent and constrained refinement
*S* = 0.99	*w* = 1/[σ^2^(*F*_o_^2^) + (0.0398*P*)^2^] where *P* = (*F*_o_^2^ + 2*F*_c_^2^)/3
3526 reflections	(Δ/σ)_max_ = 0.001
195 parameters	Δρ_max_ = 0.24 e Å^−^^3^
0 restraints	Δρ_min_ = −0.38 e Å^−^^3^

### Special details

**Table:**

Geometry. All e.s.d.'s (except the e.s.d. in the dihedral angle between two l.s. planes) are estimated using the full covariance matrix. The cell e.s.d.'s are taken into account individually in the estimation of e.s.d.'s in distances, angles and torsion angles; correlations between e.s.d.'s in cell parameters are only used when they are defined by crystal symmetry. An approximate (isotropic) treatment of cell e.s.d.'s is used for estimating e.s.d.'s involving l.s. planes.
Refinement. Refinement of *F*^2^ against ALL reflections. The weighted *R*-factor *wR* and goodness of fit *S* are based on *F*^2^, conventional *R*-factors *R* are based on *F*, with *F* set to zero for negative *F*^2^. The threshold expression of *F*^2^ > σ(*F*^2^) is used only for calculating *R*-factors(gt) *etc*. and is not relevant to the choice of reflections for refinement. *R*-factors based on *F*^2^ are statistically about twice as large as those based on *F*, and *R*- factors based on ALL data will be even larger.

### Fractional atomic coordinates and isotropic or equivalent isotropic displacement parameters (Å^2^)

**Table:**

	*x*	*y*	*z*	*U*_iso_*/*U*_eq_	
S1	0.08893 (5)	1.05001 (5)	0.25028 (4)	0.02336 (13)	
S2	0.20597 (5)	1.15671 (5)	0.45732 (4)	0.02242 (13)	
N1	0.74042 (17)	0.74849 (16)	0.19582 (13)	0.0200 (3)	
N2	0.45173 (18)	0.88209 (17)	0.12486 (14)	0.0210 (3)	
N3	0.37052 (17)	1.13818 (15)	0.25715 (13)	0.0192 (3)	
O1	0.24938 (15)	0.82797 (14)	0.02096 (13)	0.0320 (3)	
C1	0.6056 (2)	0.74785 (18)	0.12785 (15)	0.0189 (4)	
C2	0.6186 (2)	0.62959 (19)	0.06672 (16)	0.0217 (4)	
H2	0.5203	0.6332	0.0196	0.026*	
C3	0.7803 (2)	0.50655 (19)	0.07723 (16)	0.0233 (4)	
H3	0.7949	0.4229	0.0371	0.028*	
C4	0.9210 (2)	0.50511 (19)	0.14621 (16)	0.0217 (4)	
H4	1.0329	0.4214	0.1533	0.026*	
C5	0.8961 (2)	0.62787 (19)	0.20491 (16)	0.0204 (4)	
C6	1.0426 (2)	0.6313 (2)	0.28327 (18)	0.0300 (4)	
H6A	1.0281	0.7452	0.2761	0.045*	
H6B	1.1550	0.5765	0.2538	0.045*	
H6C	1.0403	0.5745	0.3711	0.045*	
C7	0.2879 (2)	0.9140 (2)	0.07486 (16)	0.0217 (4)	
C8	0.1511 (2)	1.06990 (19)	0.09143 (16)	0.0222 (4)	
H8A	0.0453	1.1009	0.0339	0.027*	
H8B	0.1975	1.1594	0.0676	0.027*	
C9	0.2379 (2)	1.11504 (18)	0.31235 (16)	0.0193 (4)	
C10	0.4590 (2)	1.19559 (18)	0.32980 (15)	0.0184 (4)	
C11	0.3900 (2)	1.21228 (18)	0.44347 (16)	0.0194 (4)	
C12	0.4660 (2)	1.26537 (19)	0.52672 (16)	0.0239 (4)	
H12	0.4194	1.2750	0.6041	0.029*	
C13	0.6122 (2)	1.3037 (2)	0.49261 (17)	0.0257 (4)	
H13	0.6667	1.3405	0.5476	0.031*	
C14	0.6811 (2)	1.2894 (2)	0.37940 (17)	0.0255 (4)	
H14	0.7806	1.3181	0.3583	0.031*	
C15	0.6076 (2)	1.23436 (19)	0.29716 (16)	0.0225 (4)	
H15	0.6563	1.2231	0.2207	0.027*	
H2A	0.463 (2)	0.951 (2)	0.1624 (17)	0.027 (5)*	

### Atomic displacement parameters (Å^2^)

**Table:**

	*U*^11^	*U*^22^	*U*^33^	*U*^12^	*U*^13^	*U*^23^
S1	0.0181 (2)	0.0224 (2)	0.0319 (3)	−0.00785 (18)	0.00497 (18)	−0.0107 (2)
S2	0.0215 (3)	0.0227 (2)	0.0215 (2)	−0.00602 (18)	0.00643 (18)	−0.00652 (18)
N1	0.0180 (7)	0.0194 (7)	0.0224 (8)	−0.0055 (6)	0.0019 (6)	−0.0072 (6)
N2	0.0166 (8)	0.0207 (7)	0.0274 (8)	−0.0035 (6)	−0.0001 (6)	−0.0139 (7)
N3	0.0175 (8)	0.0178 (7)	0.0215 (7)	−0.0049 (6)	0.0015 (6)	−0.0062 (6)
O1	0.0229 (7)	0.0330 (7)	0.0440 (8)	−0.0057 (5)	−0.0046 (6)	−0.0229 (7)
C1	0.0186 (9)	0.0183 (8)	0.0186 (8)	−0.0054 (7)	0.0036 (7)	−0.0052 (7)
C2	0.0220 (9)	0.0220 (8)	0.0237 (9)	−0.0086 (7)	0.0046 (7)	−0.0095 (7)
C3	0.0281 (10)	0.0191 (8)	0.0246 (9)	−0.0088 (7)	0.0078 (8)	−0.0094 (7)
C4	0.0206 (9)	0.0156 (8)	0.0250 (9)	−0.0032 (7)	0.0070 (7)	−0.0042 (7)
C5	0.0187 (9)	0.0191 (8)	0.0207 (9)	−0.0056 (7)	0.0046 (7)	−0.0030 (7)
C6	0.0215 (10)	0.0271 (9)	0.0374 (11)	−0.0034 (8)	−0.0007 (8)	−0.0097 (9)
C7	0.0178 (9)	0.0236 (8)	0.0230 (9)	−0.0056 (7)	0.0016 (7)	−0.0082 (8)
C8	0.0179 (9)	0.0229 (9)	0.0252 (9)	−0.0046 (7)	−0.0016 (7)	−0.0096 (8)
C9	0.0185 (9)	0.0137 (7)	0.0211 (9)	−0.0005 (6)	0.0015 (7)	−0.0047 (7)
C10	0.0198 (9)	0.0142 (7)	0.0188 (8)	−0.0036 (6)	−0.0017 (7)	−0.0041 (7)
C11	0.0194 (9)	0.0147 (8)	0.0199 (9)	−0.0023 (6)	0.0010 (7)	−0.0033 (7)
C12	0.0284 (10)	0.0212 (8)	0.0178 (9)	−0.0029 (7)	−0.0002 (7)	−0.0068 (7)
C13	0.0289 (10)	0.0201 (8)	0.0270 (10)	−0.0068 (7)	−0.0042 (8)	−0.0076 (8)
C14	0.0255 (10)	0.0233 (9)	0.0284 (10)	−0.0115 (8)	−0.0009 (8)	−0.0046 (8)
C15	0.0216 (9)	0.0241 (9)	0.0208 (9)	−0.0081 (7)	0.0043 (7)	−0.0049 (7)

### Geometric parameters (Å, °)

**Table:**

S1—C9	1.7462 (17)	C4—H4	0.9500
S1—C8	1.8133 (18)	C5—C6	1.502 (2)
S2—C11	1.7444 (17)	C6—H6A	0.9800
S2—C9	1.7459 (17)	C6—H6B	0.9800
N1—C5	1.340 (2)	C6—H6C	0.9800
N1—C1	1.345 (2)	C7—C8	1.519 (2)
N2—C7	1.359 (2)	C8—H8A	0.9900
N2—C1	1.404 (2)	C8—H8B	0.9900
N2—H2A	0.863 (17)	C10—C11	1.402 (2)
N3—C9	1.297 (2)	C10—C15	1.402 (2)
N3—C10	1.395 (2)	C11—C12	1.389 (2)
O1—C7	1.2212 (19)	C12—C13	1.385 (2)
C1—C2	1.388 (2)	C12—H12	0.9500
C2—C3	1.381 (2)	C13—C14	1.393 (3)
C2—H2	0.9500	C13—H13	0.9500
C3—C4	1.383 (2)	C14—C15	1.381 (2)
C3—H3	0.9500	C14—H14	0.9500
C4—C5	1.389 (2)	C15—H15	0.9500
			
C9—S1—C8	100.19 (8)	O1—C7—C8	121.40 (15)
C11—S2—C9	88.51 (8)	N2—C7—C8	113.96 (14)
C5—N1—C1	117.98 (13)	C7—C8—S1	113.25 (12)
C7—N2—C1	128.33 (14)	C7—C8—H8A	108.9
C7—N2—H2A	116.3 (12)	S1—C8—H8A	108.9
C1—N2—H2A	115.3 (12)	C7—C8—H8B	108.9
C9—N3—C10	110.02 (14)	S1—C8—H8B	108.9
N1—C1—C2	123.80 (15)	H8A—C8—H8B	107.7
N1—C1—N2	111.89 (13)	N3—C9—S2	116.86 (12)
C2—C1—N2	124.31 (15)	N3—C9—S1	124.62 (13)
C3—C2—C1	117.24 (15)	S2—C9—S1	118.51 (10)
C3—C2—H2	121.4	N3—C10—C11	115.18 (15)
C1—C2—H2	121.4	N3—C10—C15	124.86 (16)
C2—C3—C4	119.97 (15)	C11—C10—C15	119.96 (15)
C2—C3—H3	120.0	C12—C11—C10	121.74 (16)
C4—C3—H3	120.0	C12—C11—S2	128.85 (14)
C3—C4—C5	119.00 (16)	C10—C11—S2	109.42 (12)
C3—C4—H4	120.5	C13—C12—C11	117.45 (17)
C5—C4—H4	120.5	C13—C12—H12	121.3
N1—C5—C4	122.01 (15)	C11—C12—H12	121.3
N1—C5—C6	116.40 (14)	C12—C13—C14	121.46 (16)
C4—C5—C6	121.59 (15)	C12—C13—H13	119.3
C5—C6—H6A	109.5	C14—C13—H13	119.3
C5—C6—H6B	109.5	C15—C14—C13	121.31 (17)
H6A—C6—H6B	109.5	C15—C14—H14	119.3
C5—C6—H6C	109.5	C13—C14—H14	119.3
H6A—C6—H6C	109.5	C14—C15—C10	118.07 (16)
H6B—C6—H6C	109.5	C14—C15—H15	121.0
O1—C7—N2	124.63 (16)	C10—C15—H15	121.0
			
C5—N1—C1—C2	0.0 (3)	C11—S2—C9—N3	0.20 (13)
C5—N1—C1—N2	−179.13 (14)	C11—S2—C9—S1	−178.48 (10)
C7—N2—C1—N1	−173.97 (16)	C8—S1—C9—N3	−10.77 (15)
C7—N2—C1—C2	6.9 (3)	C8—S1—C9—S2	167.80 (9)
N1—C1—C2—C3	−0.1 (3)	C9—N3—C10—C11	1.10 (19)
N2—C1—C2—C3	178.95 (16)	C9—N3—C10—C15	−179.48 (14)
C1—C2—C3—C4	−0.2 (2)	N3—C10—C11—C12	178.84 (14)
C2—C3—C4—C5	0.5 (2)	C15—C10—C11—C12	−0.6 (2)
C1—N1—C5—C4	0.3 (2)	N3—C10—C11—S2	−0.95 (17)
C1—N1—C5—C6	−179.05 (15)	C15—C10—C11—S2	179.60 (12)
C3—C4—C5—N1	−0.6 (3)	C9—S2—C11—C12	−179.36 (15)
C3—C4—C5—C6	178.74 (16)	C9—S2—C11—C10	0.42 (12)
C1—N2—C7—O1	−1.3 (3)	C10—C11—C12—C13	0.8 (2)
C1—N2—C7—C8	178.66 (15)	S2—C11—C12—C13	−179.40 (12)
O1—C7—C8—S1	105.85 (18)	C11—C12—C13—C14	−0.1 (2)
N2—C7—C8—S1	−74.11 (17)	C12—C13—C14—C15	−0.8 (3)
C9—S1—C8—C7	91.58 (12)	C13—C14—C15—C10	1.1 (2)
C10—N3—C9—S2	−0.76 (17)	N3—C10—C15—C14	−179.76 (14)
C10—N3—C9—S1	177.84 (11)	C11—C10—C15—C14	−0.4 (2)

### Hydrogen-bond geometry (Å, °)

**Table:**

*D*—H···*A*	*D*—H	H···*A*	*D*···*A*	*D*—H···*A*
N2—H2A···N3	0.867 (18)	2.142 (18)	2.949 (2)	154.6 (16)
C2—H2···O1	0.95	2.30	2.890 (2)	119
C8—H8A···O1^i^	0.99	2.31	3.239 (2)	156
C8—H8B···N3	0.99	2.47	2.905 (2)	106
C12—H12···N1^ii^	0.95	2.57	3.498 (2)	166
C8—H8B···Cg2^iii^	0.99	2.68	3.494 (2)	140

Symmetry codes: (i) −*x*, −*y*+2, −*z*; (ii) −*x*+1, −*y*+2, −*z*+1; (iii) −*x*+1, −*y*+2, −*z*.
